# Toward Suppressing Charge Trapping Based on a Combined Driving Waveform with an AC Reset Signal for Electro-Fluidic Displays

**DOI:** 10.3390/membranes12111072

**Published:** 2022-10-29

**Authors:** Zhengxing Long, Zichuan Yi, Hu Zhang, Liming Liu, Lingling Shui

**Affiliations:** 1 College of Electron and Information, University of Electronic Science and Technology of China Zhongshan Institute, Zhongshan 528402, China; 2 School of Information and Optoelectronic Science and Engineering, South China Normal University, Guangzhou 510006, China

**Keywords:** electro-fluidic displays (EFDs), driving waveform, charge trapping, leakage current, dielectric loss power

## Abstract

Digital microfluidic technology based on the principle of electrowetting is developing rapidly. As an extension of this technology, electro-fluidic displays (EFDs) have gradually become a novel type of display devices, whose grayscales can be displayed by controlling oil film in pixels with a microelectromechanical system (MEMS). Nevertheless, charge trapping can occur during EFDs’ driving process, which will produce the leakage current and seriously affect the performance of EFDs. Thus, an efficient driving waveform was proposed to resolve these defects in EFDs. It consisted of a driving stage and a stabilizing stage. Firstly, the response time of oil film was shortened by applying an overdriving voltage in the driving stage according to the principle of the electrowetting. Then, a direct current (DC) voltage was designed to display a target luminance by analyzing leakage current-voltage curves and a dielectric loss factor. Finally, an alternating current (AC) reset signal was applied in the stabilizing stage to suppress the charge trapping effect. The experiment results indicated that compared with a driving waveform with a reset signal and a combined driving waveform, the average luminance was improved by 3.4% and 9.7%, and the response time was reduced by 29.63% and 51.54%, respectively.

## 1. Introduction

With the development of microelectronic technology, applications of microfluidic technology and microelectromechanical system (MEMS) devices in biomedicine, microreactors and optoelectronic displays are becoming more and more mature. As an extended application of digital microfluidic technology, electro-fluidic display (EFD) is a new display technology which is based on the electrowetting principle and microfluidic technology [1,2,3]. EFD realizes the ability of video display by driving oil film in micropixels [4,5,6]. Compared with traditional electrophoretic displays, it has excellent characteristics of wide viewing angle, high reflectivity, and readability [7,8,9,10]. However, leakage current in EFDs’ insulating layer can be caused by charge trapping. Thus, it is very important to suppress the charge trapping in EFDs by designing novel driving waveforms for improving display performance.

Oil film in pixels had phenomena of oscillation, splitting and charge trapping, which will cause a decrease in brightness and stability. [11,12,13,14]. In the experiment of solving oil film splitting, a driving waveform was designed by analyzing a proportional function model [15]. However, this might result in long response times. To resolve this drawback, a mixed waveform was proposed by combining a threshold voltage, a slope function model and square signal [16]. The initial voltage was gradually increased from the threshold voltage by applying the slope function model, and oil film in a pixel could be ruptured faster. Oil film dispersed after rupture could be fully combined by applying a square signal and it could be driven quickly to a corner of a pixel for achieving a fast response. In addition, maintaining a stable luminance was another important factor in improving the performance of EFDs. Due to charge trapping, it was difficult for EFDs to maintain a saturation luminance. In order to solve this problem, an improved AC reset waveform was designed [17]. This method could release the trapped charge in time and stabilize the luminance by quickly switching the voltage polarity in the AC signal. However, oil film backflow in EFDs could also be caused by charge trapping at the same time, and the luminance would be significantly reduced. Hence, a separated reset waveform was designed [18]. This waveform contained a reset stage and a grayscale stage. The trapped charges in the insulating layer could be efficiently released by applying the reset stage. Then, the oil film in a pixel was driven by a high voltage to make it shrink stably in the grayscale stage, which could be achieved the purpose of suppressing oil film backflow. All the above experimental results provide a reference direction for our waveform optimization research and design.

In this paper, based on the analysis of charge trapping and leakage current theory, a new AC reset driving waveform was designed for EFDs. Oil film could be ruptured rapidly to reduce the response time by applying an overdriving voltage. Next, the EFD was driven by a DC voltage to fully combine the dispersed foil film. Finally, the AC reset signal was designed according to the leakage current theory. By reducing charge trapping in the insulation layer, the leakage current could be reduced and the display stability could be improved.

## 2. Principles and Methods

### 2.1. Principle of EFDs

**Figure 1:**
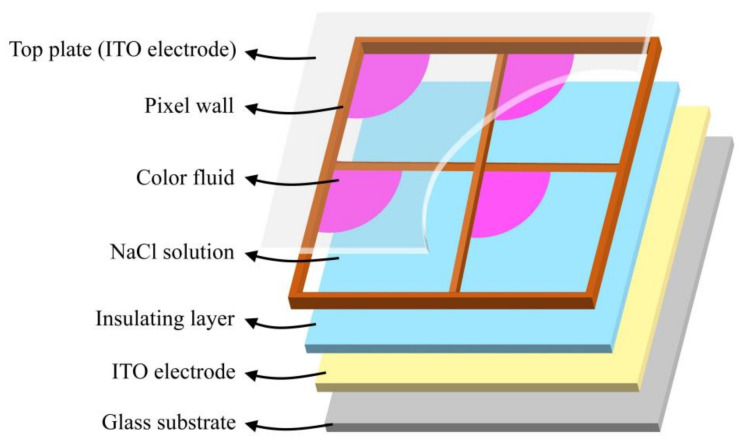
The spatial structure of an EFD in the shrinkage state of oil film. It is composed of a top plate, pixel walls, NaCl solution, color oil film, an insulating layer, two ITO electrodes and a glass substrate. The pixel shows the color of the substrate when a voltage is applied.

**Figure 2:**
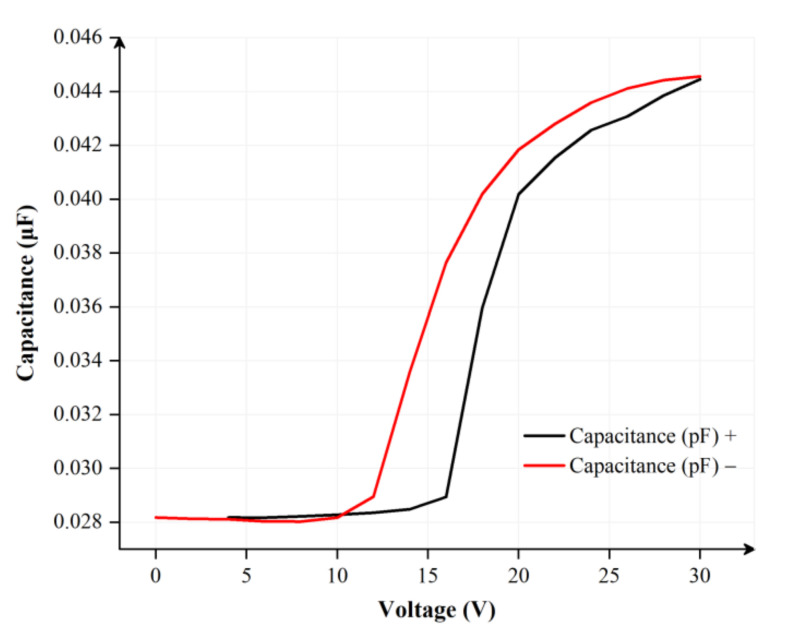
The C-V curve of EFDs within 500 ms. The voltage change rate was ±0.06 V/ms. There was a significant hysteresis between two C-V curves for positive and negative voltages.

A voltage can be applied between two ITO electrodes in EFDs to change the wettability of material surface, which can control the movement of oil film [19,20]. The composition of a single pixel in EFDs mainly includes a glass substrate, pixel walls, an insulating layer, polar oil film (NaCl solution), colored oil film and two indium tin oxide (ITO) electrodes [21,22,23], as shown in Figure 1. When the driving voltage of a pixel is 0, oil film spreads naturally and covers the whole pixel; the EFD shows the color of the oil film. As the voltage applied to the pixel gradually increases, the equilibrium state of the oil film in the pixel is destroyed, and the oil film begins to split and move closer to the pixel walls until it is fully gathered. At this time, the glass substrate is exposed and EFD displays the color of the substrate. The luminance display in a pixel depends on the area occupied by the shrinkage oil film [24,25,26,27]. In addition, the contact angle is an important factor to characterize the degree of oil film shrinkage, which is usually calculated with the Lippmann–Young equation, as shown in Equation (1) [6]:

(1) $$\cos\theta_{x}=\cos\theta_{0}+\frac{\varepsilon_{v}\varepsilon_{il}}{2d\gamma_{Ol}}V^{2}$$

(2) $$V_{d}=\sqrt{\frac{2\left(1-\cos\theta \right)\gamma_{Ol}}{\frac{1}{4\pi k}\frac{\varepsilon_{oil}S_{oil}}{h}+\frac{\varepsilon_{il}S_{pix}}{d}}}\sim \sqrt{d}$$

where $\theta_{x}$ represents a dynamic contact angle, $\theta_{0}$ indicates a static contact angle, $\varepsilon_{v}$ and $\varepsilon_{il}$ are dielectric constants of a vacuum and an insulating layer, respectively, d represents the thickness of the insulating layer, $\gamma_{Ol}$ is the interfacial tension between different oil films, V indicates the voltage applied between two ITO electrodes, $V_{d}$ represents the dielectric breakdown voltage, $\varepsilon_{oil}$ is the dielectric constant of the oil film, h indicates the height of the oil film and $S_{oil}$ and $S_{pix}$ correspond to the shrinkage area of the oil film and the area of an entire pixel, respectively. It can be seen from Equation (2) [9] that the applied voltage cannot exceed the dielectric breakdown voltage of the insulating layer; otherwise, the insulating layer will be destroyed and the performance of EFDs will be impaired. In addition, the C-V curve of the EFD collected during a certain time is shown in Figure 2. The equivalent capacitance of the insulating layer tends to rise when the initial voltage is increased to exceed the threshold voltage according to a predetermined step value. However, the change of the C-V curve is not linear, and there is a significant hysteresis between the rising stage and falling stage.

### 2.2. Charge Trapping of EFDs

**Figure 3:**
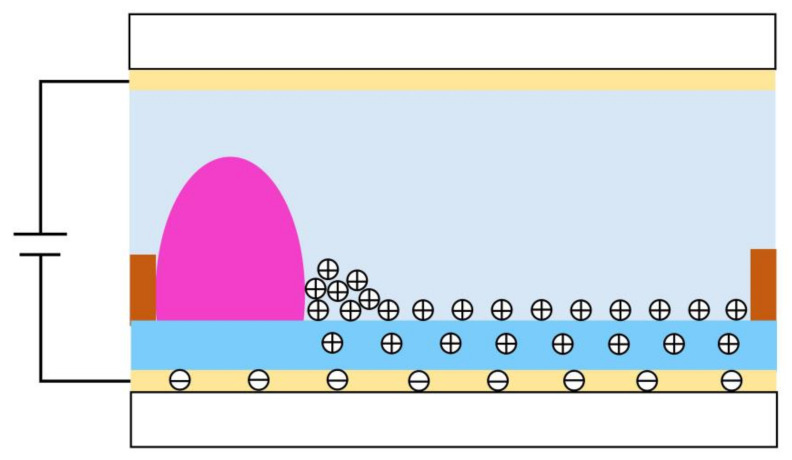
The phenomenon of charge trapping. Part of charge is trapped in the insulating layer when a voltage is applied between two ITO electrodes, resulting in the formation of a reverse electric field. The charge trapping phenomenon occurred when trapped charges cannot be released efficiently.

Due to a continuous action of the applied voltage, the charged particles are trapped in the insulating layer, which leads to the charge trapping effect, as shown in Figure 3 [28,29]. The contact angle of oil film in EFDs can be affected by the charge trapping effect. In addition, the electrowetting performance depends only on the amplitude of the driving voltage rather than the polarity, according to the Lippmann–Young equation. However, according to the existing experimental results, the change of the contact angle in EFDs is also related to the voltage polarity [30]. Therefore, the Lippmann–Young equation must be modified on the original basis, as shown in Equation (3) [31]:

(3) $$\cos\theta_{x}=\cos\theta_{0}+\frac{\varepsilon_{v}\varepsilon_{il}}{2d\gamma_{Ol}}{\left(V-V_{s}\right)}^{2}$$

where $V_{s}$ is the potential generated by the charge trapping effect. During the driving process, most of charges can be accumulated near three-phase contact lines, and a small number of charges are trapped. Therefore, the effect of the electric field force is weakened. With the increase of driving time, the charge accumulated in the insulation layer is also increasing, and the oil film gradually diffuses to the whole pixel. 

### 2.3. Leakage Current of EFDs

**Figure 4:**
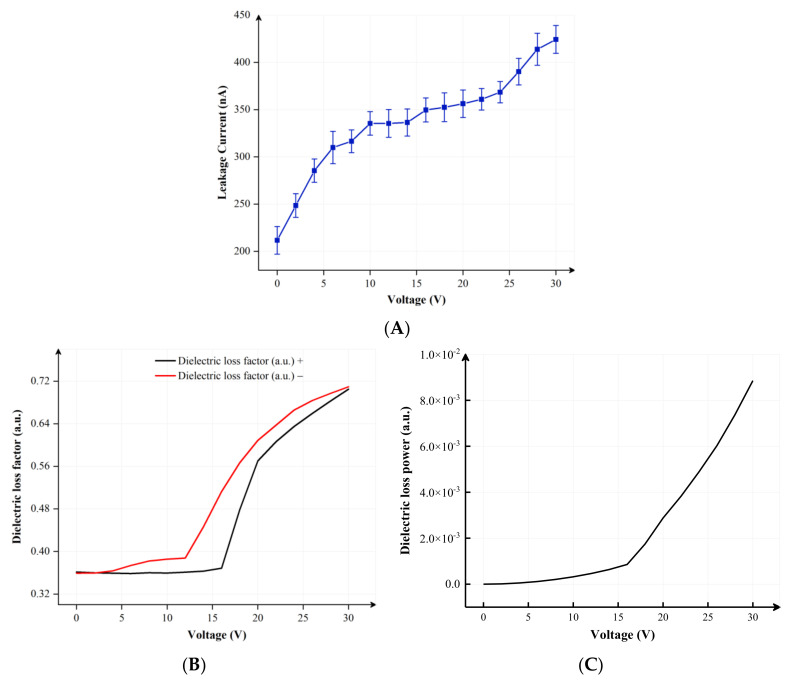
Measurement of performance parameters for EFDs. (**A**) The change curve of leakage current in EFDs. The measurement rate was 2 V/s. Error bars were obtained by repeating the experiment three times. (**B**) The dielectric loss factor curve of EFDs within 500 ms. The black line and the red line represented the rising curve and the falling curve, respectively. (**C**) Luminance curves of dielectric loss power in different V. The dielectric loss power was positively correlated with $V_{max}$, and it changed in real time with V.

Due to the effect of charge trapping, a leakage current is generated inside EFDs, as shown in Figure 4A [32]. At the same time, the electric field will produce dielectric loss. When driving voltages are lower than the partial discharge voltage, the dielectric loss in the insulation layer is only caused by the leakage current [33,34]. However, when an AC voltage is applied between two ITO electrodes, the capacitance current will appear in EFDs. Due to the dielectric loss, the total current in EFDs can be calculated by Equation (4):

(4) $$i=i_{c}+i_{r}$$

where i is the total current, $i_{c}$ is the capacitance current and $i_{r}$ is the leakage current. At this point, the apparent power and dielectric loss power provided by a driving voltage can be expressed as Equation (5) and Equation (6), respectively:

(5) $$S=P+jQ=Ui_{r}+jUi_{c}$$

(6) $$P=Q\tan\delta =U^{2}\omega C\tan\delta$$

where U is the AC voltage applied between two ITO electrodes, C is the equivalent capacitance of the insulating layer, ω is the angular frequency of the voltage, δ is the dielectric loss angle, tanδ is the dielectric loss factor, as shown in Figure 4B, S represents the apparent power, which is defined as the product of the effective value of voltage and the effective value of current, which also represents the maximum dielectric loss power in a pixel, and P is the dielectric loss power, which represents the energy consumed by the dielectric in the pixel in a unit of time. It is positively related to driving voltages, as shown in Figure 4C. In addition, dielectric loss power is only related to the equivalent capacitance of the insulating layer and dielectric loss factor when the applied voltage and frequency are constant. Therefore, in order to compare the influence of equivalent capacitance and dielectric loss factor on dielectric loss power, the experiment results are standardized by a multiple linear regression method [35]. The standardized regression coefficients are 0.411 and 0.556, respectively. The standardized regression coefficients of the dielectric loss factor are increased by 35.28% compared with the equivalent capacitance. In addition, the absolute value of standardized regression coefficients of the dielectric loss factor is greater than the equivalent capacitance. Therefore, the dielectric loss factor has a more significant impact on the dielectric loss power. The influence of the dielectric loss power must be fully considered when a driving waveform is designed to optimize the performance of EFDs.

### 2.4. Design of Driving Waveforms

**Figure 5:**
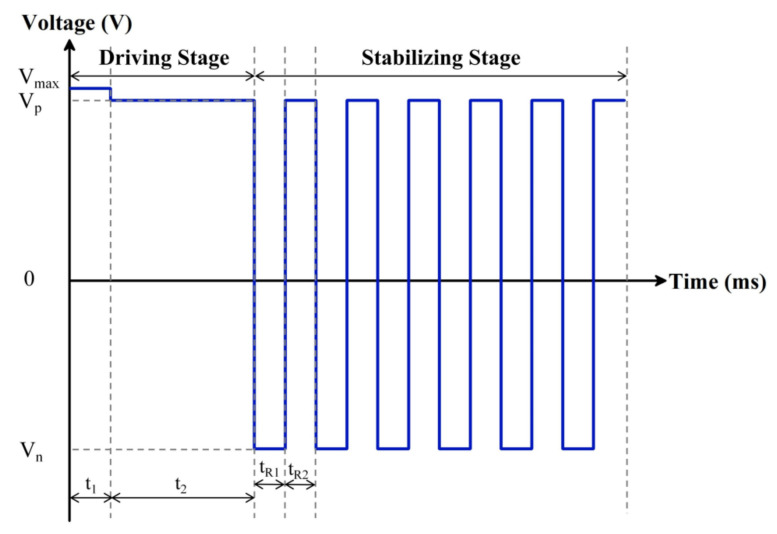
The proposed driving waveform for suppressing the charge trapping effect. It was combined with a driving stage and a stabilizing stage. The driving stage was an overdriving voltage and a DC voltage; the stabilizing stage was an AC pulse signal. Here, $V_{max}$ is the overdriving voltage of the driving stage, $V_{p}$ is the DC voltage of the driving stage, $V_{n}$ is the negative voltage of the stabilizing stage, $t_{1}$ and $t_{2}$ are the duration of the overdriving voltage and DC voltage, respectively, $t_{R1}$ is the driving time of the positive voltage in the stabilizing stage and $t_{R2}$ is the release time of the negative voltage in the stabilizing stage.

Due to the charge trapping in the insulation layer, the leakage current is generated in EFDs, and it is difficult to maintain a stable luminance display. To solve this defect, a novel type of driving waveform was proposed. It was combined with a driving stage and a stabilizing stage, as shown in Figure 5, where $V_{max}$ is an overdriving voltage which could break the oil film quickly, $V_{p}$ is a DC voltage which enabled EFDs to display a target luminance, $V_{n}$ is a negative voltage in the AC pulse signal, f is the frequency of the AC pulse signal, $t_{1}$ and $t_{2}$ are durations of the overdriving voltage and the DC voltage, respectively, and $t_{R1}$ and $t_{R2}$ are the durations of positive and negative voltages in one period of the AC pulse signal, respectively.

In the driving stage, the driving signal included an overdriving voltage and a DC voltage. The overdriving voltage was used to turn on pixels quickly. Then, the DC driving signal was introduced to fully combine or fuse oil film with other dispersed oil film, so as to achieve a target luminance. At the same time, in order to remove trapped charges in the insulating layer and reduce the influence of leakage current, an AC pulse signal was introduced in the stabilizing stage. The AC pulse signal was composed of a square signal with unequal positive and negative voltages. EFDs were driven by the positive voltage to obtain a better luminance, and the negative voltage was used to release trapped positive charges in the insulating layer to improve the display stability. Therefore, not only the generation of leakage current could be suppressed, but also the obstacle of display instability could be overcome by applying the proposed driving waveform.

## 3. Experimental Results and Discussion

### 3.1. Experimental Platform

**Figure 6:**
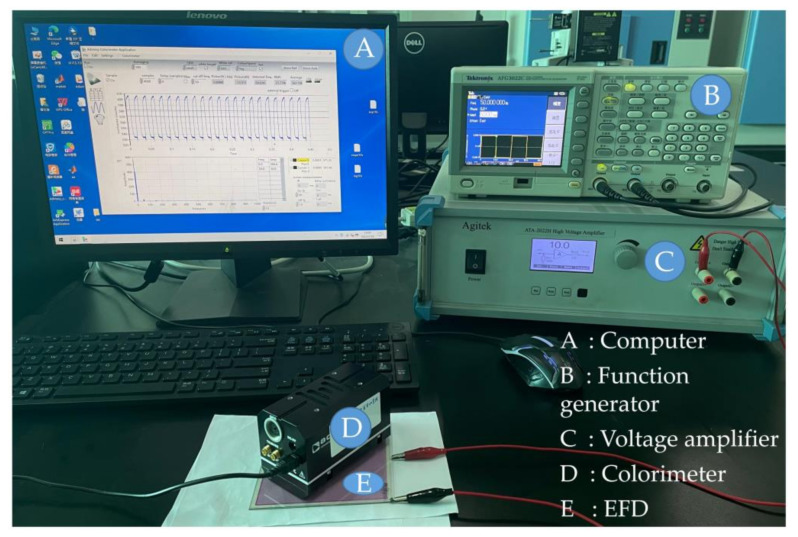
A performance verification platform for driving waveforms. It consisted of a colorimeter, an EFD, a computer, a function generator and a voltage amplifier. The computer, the function generator and the voltage amplifier were responsible for designing, amplifying and outputting the driving waveform. Next, the luminance change of EFDs was collected by the colorimeter. Then, the calculation of luminance was completed by the computer.

The experimental platform consisted of a waveform editing system, an acquisition system and an EFD, as shown in Figure 6. The waveform editing system completed the design of driving waveforms by a computer (H430, Lenovo, Beijing, China). A function generator (AFG3022C, Tektronix, Beaverton, OR, USA) and a voltage amplifier (ATA-2022H, Agitek, Xian, China) were used to amplify and output the driving waveform. The acquisition system completed the collection and analysis of EFDs’ luminance by a colorimeter (Arges-45, Admesy, Ittervoort, The Netherlands) and a computer. The specific experimental operation process was as follows.

Firstly, the designed driving waveform could be edited by using Matlab software, and Arbexpress software was used to convert the edited waveform file, which could be saved in an external storage device. Secondly, the external storage device was used to input the edited waveform file to the function generator. Next, various parameters were set in the function generator, considering the voltage range of the signal generator, the output driving voltage of the function generator needed to be amplified by the voltage amplifier, and the amplification factor was 10 times. Finally, the luminance acquisition of the EFD was carried out by using a colorimeter.

### 3.2. Testing of the Driving Stage

**Figure 7:**
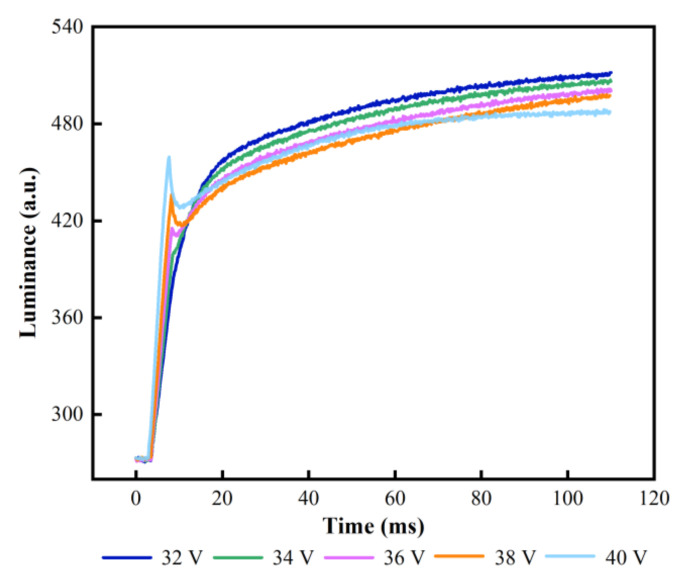
Luminance-time curves of different $V_{max}$s. Luminance-time curves of different $V_{max}$s. With the increase of $V_{max}$, the luminance gradually decreased and luminance mutations of varying degrees begin to appear. The maximum luminance was 511 when $V_{max}$ was 32 V.

In the experiments, $V_{max}$ was set to 32–40 V and $t_{1}$ was set to 4 ms. Luminance change curves of different $V_{max}$s are shown in Figure 7. It can be seen that the initial luminance before driving was 272. The rising rate of luminance and the maximum luminance were decreasing gradually when $V_{max}$ was increased. This was because more charged particles were trapped when the upper limit of $V_{max}$ was increased, which would result in an increase of the leakage current in EFDs. At the same time, the capacitance value and dielectric loss factor in the pixel also increased. Thus, the dielectric loss power would be increased with the increase of $V_{max}$. In order to reduce the influence of the leakage current and the dielectric loss power, $V_{max}$ must be adjusted reasonably. The highest luminance was 511 when $V_{max}$ was 32 V. At this time, the leakage current generated by the charge trapping had minimal effect on the performance of EFDs. Therefore, $V_{max}$ was set to 32 V for obtaining the highest luminance.

**Figure 8:**
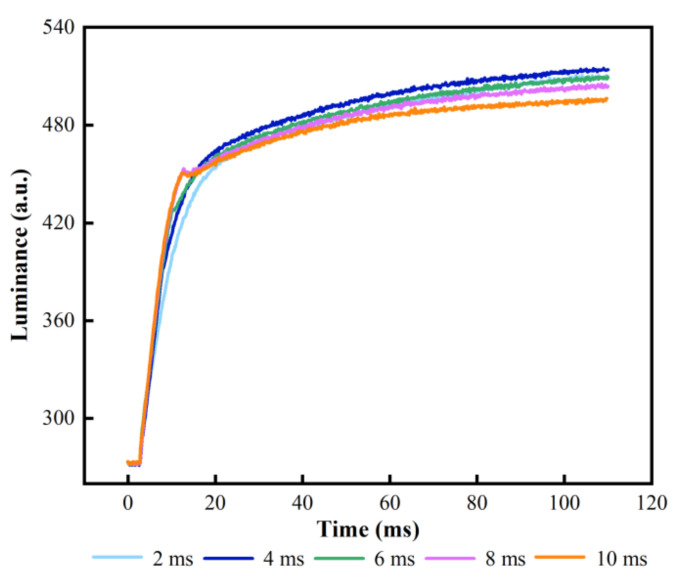
Luminance curves of different $t_{1}$s. Luminance is proportional to the duration of the overdriving voltage in the driving stage. The highest luminance was 514 when $t_{1}$ was 4 ms.

At the same time, several time intervals were measured to select the optimum overdriving time. In the driving stage, $V_{max}$ was set to 32 V and $t_{1}$ was set to 2–10 ms. Luminance rising curves of different $t_{1}$ are shown in Figure 8. The rising speed of luminance was decreasing gradually when $t_{1}$ was increased. However, the luminance began to decrease when $t_{1}$ was greater than 4 ms. This was because the pixel was already fully turned on when $t_{1}$ was 4 ms. However, more charges would be trapped, which could reduce the saturation luminance of EFDs when $t_{1}$ was exceed 4 ms. The highest luminance was 514 when $t_{1}$ was 4 ms. Therefore, $t_{1}$ should be set to 4 ms.

**Figure 9:**
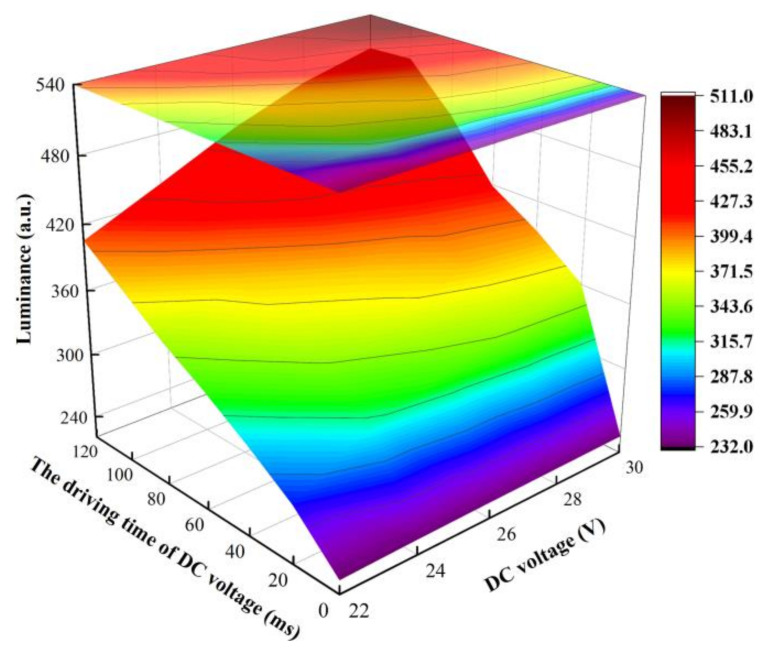
The luminance was changed with different $V_{p}$s and $t_{2}$s in the proposed driving waveform. A maximum luminance could be gained when $V_{p}$ and $t_{2}$ were set to 30 V and 100 ms, respectively.

In addition, the influence of the DC voltage and the driving time were analyzed, respectively. The selection range of $V_{p}$ and $t_{2}$ were 22–30 V and 0–120 ms, respectively. Luminance curves of different $V_{Fn}$s and $t_{2}$s are shown in Figure 9. It can be observed that the overall change of luminance showed an upward trend. The upper limit of luminance was gradually augmented and tended to saturate when $V_{p}$ was 30 V. It could be proven that the oil film could be fully combined by applying a long DC driving time. When $V_{p}$ was 30 V and $t_{2}$ was 100 ms, the maximum value of the luminance was 510. Therefore, $V_{p}$ and $t_{2}$ were set to 30 V and 100 ms, respectively.

### 3.3. Testing of the Stabilizing Stage

**Figure 10:**
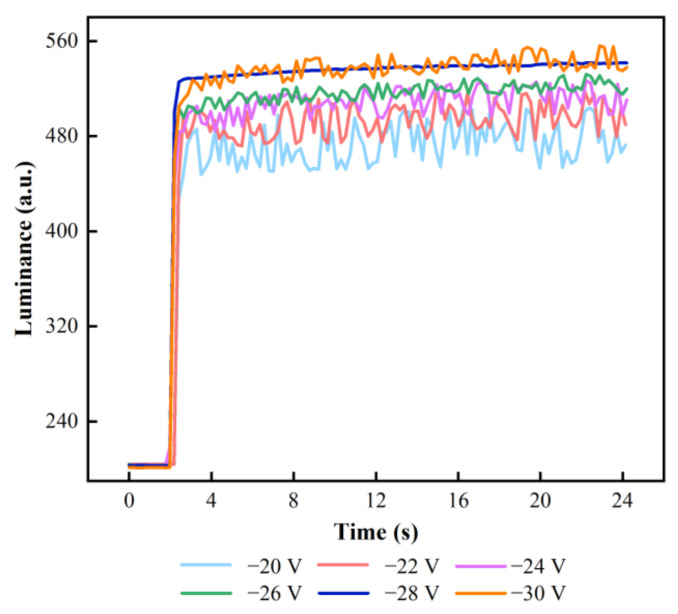
Luminance-time curves of EFDs driven by AC pulse signal with different $V_{n}$s. With the increase of negative voltage in the stabilizing stage, the degree of luminance oscillation gradually decreased, and the charge trapping phenomenon could be effectively suppressed. The luminance curve was most stable when $V_{n}$ was −28 V.

In the testing of stabilization stage, f of the AC pulse signal was set to 50 Hz. The variation trend between $\;V_{n}$ and luminance is shown in Figure 10. As the voltage decreased, the luminance oscillation became more severe when $V_{n}$ was less than −28 V. The EFD could not maintain a stable display of luminance. This was because a low reverse pulse signal was not conducive to the sufficient release of trapped charges. Therefore, $V_{n}$ was set to −28 V by analyzing the measured results.

**Figure 11:**
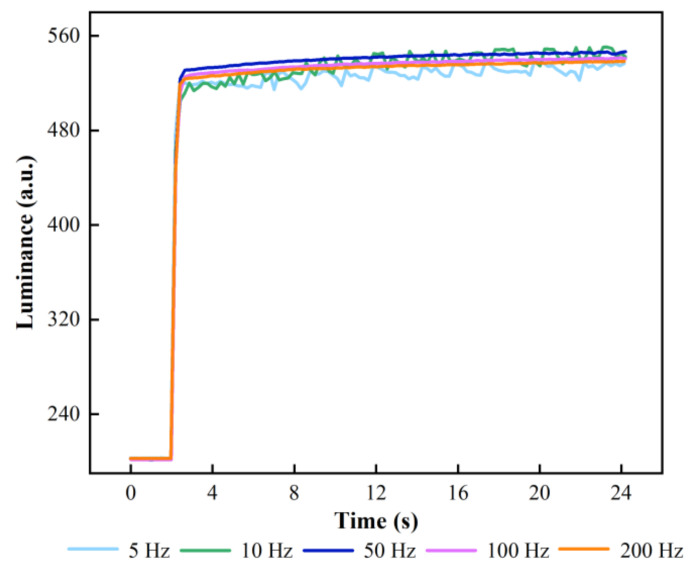
Luminance curves with different fs of the stabilizing stage in the proposed driving waveform. The luminance curve showed a severe oscillation when f was less than 50 Hz. The saturation luminance of EFDs was decreased when f was greater than 50 Hz. This is because the short duration of the negative voltage in the AC pulse signal, trapped charges in the insulating layer could not be released in time. The saturation luminance was maximum and the luminance curve had no significant oscillation when f was 50 Hz.

In addition, luminance oscillations could also cause flickering, which severely affected the visual experience. The flicker in EFDs was capable of being suppressed by selecting the best f. Luminance variation curves of EFDs driven by five different fs are shown in Figure 11. In the stabilizing stage, f was set to 5, 10, 50, 100 and 200 Hz, respectively. When f was less the 50 Hz, the luminance curve had a significant oscillation. When f exceeded 50 Hz, the luminance curve tended to be flattened, and flickers could not be observed on EFDs. However, the luminance gradually decreased with the increase of f. The reason was that the reverse electric field could be formed when the AC pulse signal was a negative voltage. Therefore, the charge in the insulation layer could be effectively released, but it could not be completely released due to a short duration. The maximum luminance was 546 when f was 50 Hz. Therefore, f was set to 50 Hz.

### 3.4. Performance Comparison

**Figure 12:**
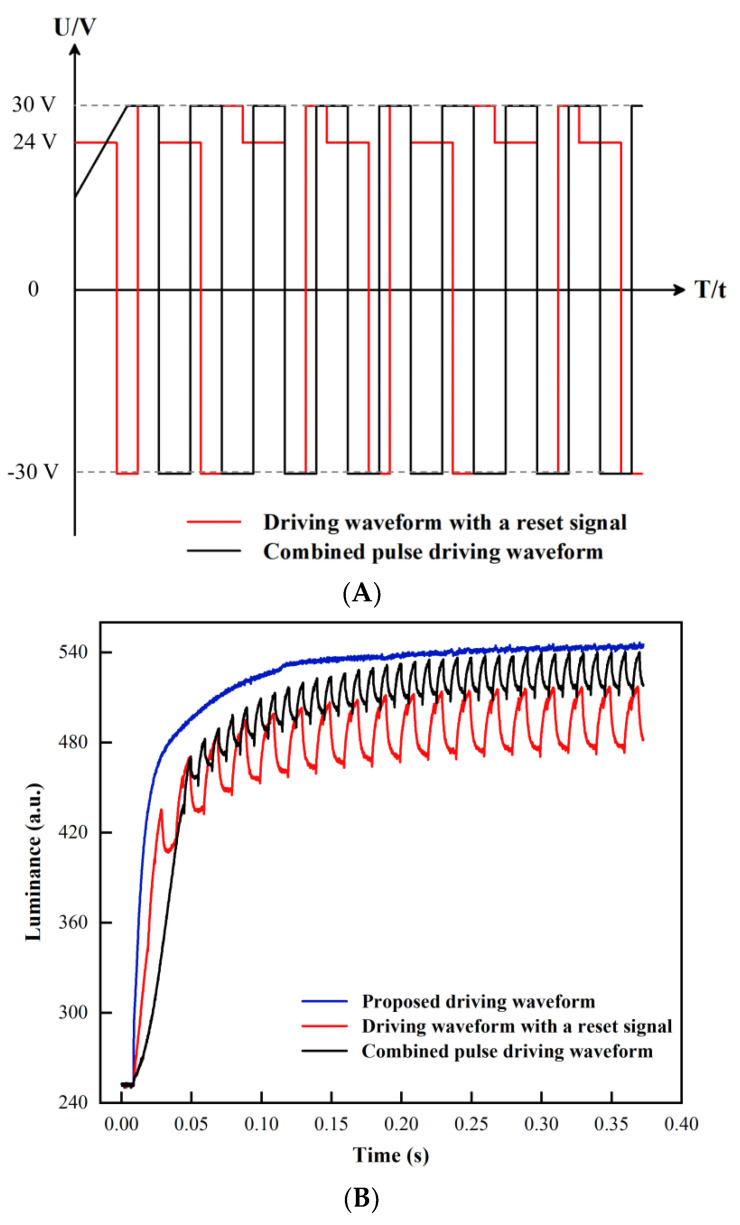

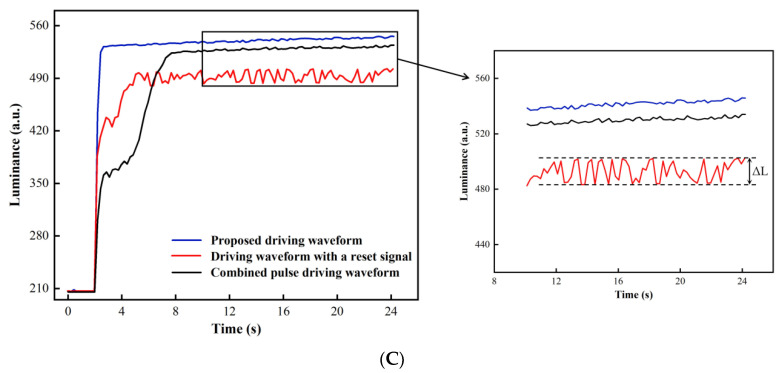
Different driving waveforms for performance comparison. (**A**) Two driving waveforms were used for performance comparison. (**B**) Luminance-time curves of three different driving waveforms. The luminance oscillation would not be produced and a higher saturation luminance could be obtained by the proposed driving waveform in the driving stage. An obvious luminance oscillation would be produced by the other two comparison driving waveforms. (**C**) Luminance variation curves of three different driving waveforms within 24 s. The driving waveform with a reset signal would generate luminance oscillation with an amplitude of ΔL. However, the phenomenon of charge trapping could be solved effectively and luminance oscillation could be reduced to achieve a stable display of luminance by the proposed driving waveform.

The performance comparison of three different driving waveforms is shown in Figure 12A. The two driving waveforms involved in the comparison included a driving waveform with a reset signal [17] and a combined pulse driving waveform [16]. The positive voltage amplitude, negative voltage amplitude and frequency of the waveform with a reset signal were 30 V, −30 V and 50 Hz, respectively. Parameters of the combined pulse driving waveform were the same. 

Luminance rising curves of three different driving waveforms in the driving stage are shown in Figure 12B. It can be observed that the average luminance was 543 when the EFD was driven by the proposed waveform, higher than the other two comparison driving waveforms in the same measurement period. This was because the overdriving voltage increased the electric field force, and oil film was broken quickly by the driving of the electric field force. Then, this dispersed oil film was fully combined and shrunk to a corner of a pixel by the driving of DC voltage to improve the luminance of EFDs. In addition, it could be shown from the rising curve of luminance that the luminance of two comparison driving waveforms generated periodic oscillation when the luminance reached 490 and 515, respectively. The average luminance of the driving waveform with a reset signal and the combined pulse driving waveform were 495 and 525, respectively. In addition, the response time of the driving waveform with a reset signal was 130 ms and the response time of the combined pulse driving waveform was 108 ms. On the contrary, the oil film could be shrunk fully in only 76 ms when the EFD was driven by the proposed driving waveform. Therefore, the average luminance was increased by 9.7% compared with the driving waveform with a reset signal and the response time was reduced by 29.63% compared with the combined pulse driving waveform. Moreover, the luminance change curve of the oil film in the stabilizing stage could be observed in Figure 12C. The trapped charge could be released in time to improve the luminance oscillation by applying the proposed driving waveform. Thus, the luminance curve of the proposed driving waveform showed no obvious change. However, the luminance curve would produce a luminance oscillation with an amplitude of ΔL when it was driven by the driving waveform with a reset signal, and the EFD could not maintain a stable display of luminance. In addition, luminance oscillation could be improved when it was driven by the combined pulse driving waveform, but response times were increased and luminance was decreased. It was proven that the proposed driving waveform could not only greatly shorten the response time of EFDs, but also released the trapped charges in time through the AC reset signal to solve the defect of luminance oscillation and oil film backflow. 

## 4. Conclusions

In this paper, a novel driving waveform based on the principle of charge trapping and leakage current was proposed to reduce the effect of charge trapping in EFDs. Firstly, oil film dispersion was effectively reduced and the response time of the oil film was shortened by optimizing the measurement parameters of an overdriving voltage and a DC voltage in the driving stage. Then, the luminance oscillation was declined and the saturation luminance was improved by applying an AC reset signal in the stabilizing stage. Compared with two different driving waveforms, the effect of the leakage current and luminance oscillation in oil film could be effectively decreased by the proposed driving waveform. As a whole, we designed a driving waveform for improving the saturation luminance and display the stability of EFDs by suppressing charge trapping, which provided a certain research value for the driving design of microfluidic and MEMS.

## Data Availability

Data are contained within the article.
